# Effect of intra-articular corticosteroid injections for knee osteoarthritis on the rates of subsequent knee replacement and post-operative outcomes: a national cohort study of England

**DOI:** 10.1186/s12916-025-04000-6

**Published:** 2025-04-07

**Authors:** Samuel Hawley, Albert Prats-Uribe, Gulraj S. Matharu, Antonella Delmestri, Daniel Prieto-Alhambra, Andrew Judge, Michael R. Whitehouse

**Affiliations:** 1 Musculoskeletal Research Unit, Translational Health Sciences, Bristol Medical School, University of Bristol, Learning & Research Building Level 1, Bristol, BS10 5NB UK; 2 Centre for Statistics in Medicine, Nuffield, Department of Orthopaedics, Rheumatology and Musculoskeletal Sciences, University of Oxford, Botnar Research Centre, Oxford, OX3 7LD UK; 3 National Institute for Health Research (NIHR) Bristol Biomedical Research Centre, University Hospitals Bristol and Weston NHS Foundation Trust and University of Bristol, Bristol, UK

**Keywords:** Osteoarthritis, Steroid injection, Pain, Knee replacement, Surgery, Post-operative outcomes, Complications, Infection, Pharmacoepidemiology, Real-world evidence

## Abstract

**Background:**

Intra-articular corticosteroid injection (IACI) is an established treatment option for uncontrolled pain in osteoarthritis. There is a lack of longer-term follow-up in most studies of the effects of IACI, meaning there is scarcity of data on the impact of IACI on the subsequent need for joint replacement. Our aim was to assess the effect of IACI for knee osteoarthritis on the subsequent incidence of knee replacement surgery and on associated post-operative outcomes.

**Methods:**

We conducted a cohort study of knee osteoarthritis patients registered in the Clinical Practice Research Datalink (CPRD) GOLD database with an incident diagnosis between 2005 and 2019. Exposure was single or repeated IACI use, analysed separately. The primary outcome was knee replacement during 1-year and 5-year follow-ups. Secondary outcomes included post-operative patient-reported outcome measures and adverse events. Primary analyses used general practitioner practice preference for IACI as an instrumental variable given this methodology can account for strong and unmeasured confounding. Secondary analyses used propensity score matching, accounting for measured covariates only.

**Results:**

During 1-year follow-up, 1628/33,357 (4.9%) knee osteoarthritis patients underwent knee replacement, for which single IACI was associated with lower risk, which persisted to 5-year follow-up (incidence rate ratio: 0.52 [0.36, 0.77]). Conversely, in secondary propensity score analyses no association was found between IACI use and knee replacement rate at 1-year follow-up, and an estimated increased rate of knee replacement at 5-year follow-up. Use of IACI pre-joint replacement was not associated with any adverse post-operative outcomes, for example, 1-year complication rates (per 100 person-years) following knee replacement were 4.6 (3.8, 5.8), 4.0 (2.7, 6.0) and 5.0 (3.1, 8.1) among patients with no, single and repeat pre-joint replacement IACI use, respectively.

**Conclusions:**

Findings from our main analysis suggest that short-term pain reduction following IACI for knee osteoarthritis may translate to lower rates of knee replacement over 5 years follow-up, although contradictory associations were observed in secondary analyses which likely reflected residual confounding by indication. Reassuringly, IACI use before knee replacement was not associated with post-operative adverse outcomes.

**Supplementary Information:**

The online version contains supplementary material available at 10.1186/s12916-025-04000-6.

## Background

Osteoarthritis is an irreversible musculoskeletal condition associated with pain, morbidity, functional decline, and reduced quality of life [1–3]. It is a significant public health problem [2, 4] and leads to substantial healthcare system and societal costs [5].

In the United Kingdom (UK), osteoarthritis is mainly managed by general practitioners (GPs) in primary care, with patients proceeding to secondary (hospital) care for surgical management if considered necessary. Following a diagnosis of osteoarthritis, there is an estimated 30% average lifetime risk of knee replacement [6]. As the prevalence of osteoarthritis is increasing [7], the need for joint replacement surgery is expected to increase considerably over the coming decades [8, 9]. Intermediate surgical procedures such as arthroscopy and debridement are also routinely offered [10], although these have been discouraged given growing evidence of lack of benefit and potential harms [10, 11].

The National Institute for Health and Care Excellence (NICE) guidance on the management of osteoarthritis states referral for joint replacement should only be made after trying all other appropriate treatments [13]. Intra-articular corticosteroid injection (IACI) is an established treatment option for providing short-term pain relief and can be considered when other treatments are ineffective or unsuitable [1, 12]. NICE and Cochrane state there is no evidence of IACI therapeutic benefit on pain lasting beyond the short-term (3–6 months) [12, 13], although high heterogeneity of evidence is noted [13–15]. There is a lack of longer-term follow-up in most studies of the effects of IACI, meaning there is scarcity of data on the impact of IACI on outcomes, including the subsequent need for joint replacement [16–18]. Whilst the pain relief afforded by IACI could be expected to delay the need for surgical management, there are emerging reports of recurrent use of IACI being associated with adverse outcomes such as radiological osteoarthritis progression and joint damage [19, 20], although inconsistent findings have been reported [21, 22].

There have also been some reports of patients who receive an IACI prior to knee replacement surgery being at increased risk of post-operative infection [23], although findings have not been consistently replicated and more research is needed [24].

Our aim was therefore to use quasi-experimental methods applied to a large primary and secondary care linked electronic health record database to assess the effects of IACI for knee osteoarthritis on the incidence of subsequent joint surgery and on post-operative patient-reported outcomes and adverse events.

## Methods

### Data and participants

We obtained and used English primary care health data from the Clinical Practice Research Datalink (CPRD) GOLD [25] for the period 01/01/2005 to 28/08/2020. CPRD GOLD contains electronic primary care health records capturing data on patient demographics, symptoms, referrals, test results, diagnoses, clinical measurements, and prescribed medicines. Clinical data are entered into the database by general practice staff in the form of Read codes—a hierarchical classification system that’s been in use for over 30 years. At the time of data extraction (September 2020), CPRD GOLD covered 4.8% of the UK population, and cumulatively contained data covering approximately 19 million current and historical people from over 900 GP practices spread across the country. All-cause mortality data were linked to the Office for National Statistics (ONS) and Index of Multiple Deprivation (IMD) based on the Lower Layer Super Output Area of the patient’s postcode. Secondary care Hospital Episode Statistics (HES) data were also linked, containing hospital diagnoses and procedures for admitted patient care (APC), and associated Patient Reported Outcome Measures (PROMs). Curator software was used to perform pre-analytical data curation [26].

Incident knee osteoarthritis patients diagnosed between 01/01/2005 and 31/12/2019 were identified using Read code lists (Additional File 1: Table S1). Only patients whose data quality was flagged as acceptable for clinical research and registered at a GP practice with at least 1 year of ‘up to standard’ (the date of which is defined by CPRD in the dataset) clinical records were included [25]. People with osteoarthritis at other anatomical joints were excluded as it was otherwise difficult to confidently ascertain which joint received an IACI from the Read code data. Further exclusion criteria, measured at knee osteoarthritis diagnosis date, were: age < 20 years old, body mass index (BMI) < 15 kg/m [2], prior referral to orthopaedic surgery (given it could be reasonably expected that in these cases injection may be being offered as a pain management strategy during the waiting period for joint replacement), prevalent inflammatory arthritis, and patients for whom HES APC linkage was not available.

### Exposure

The exposure of interest was IACI. This was defined using a Read code for IACI in the knee joint or in an unspecified joint location (given the preponderance of such codes and the exclusion of patients with osteoarthritis affecting other sites).

### Outcomes

The primary outcome was knee replacement procedures, identified using Office of Population Censuses and Surveys Version 4 codes. Secondary outcomes were arthroscopy or debridement surgical procedures. Further secondary outcomes among patients who underwent knee replacement were: post-operative complications (composite outcome of stroke, myocardial infarction, pulmonary embolism, deep vein thrombosis and wound or prosthetic joint infection), and re-operations (composite outcome including debridement, manipulation under anaesthetic, and revision). We opted to use composite post-operative outcomes for formal comparisons given the anticipated sample size of the knee replacement cohort, but used each post-operative outcome individually for descriptive summaries. We also analysed PROMs, including the EuroQoL (EQ-5-D) utility index ranging from − 0.59 (worst state) to 1.00 (best state), which is a quality of life measure across five sub-scales: mobility, self-care, daily activities, pain or discomfort, and depression or anxiety [27]. Oxford Knee Score (OKS) was also examined, which is an instrument used to measure joint pain and function from the patient’s perspective with 12 questions each with five possible responses (each scored 0 to 4), giving a total score ranging from 0 (worst) to 48 (best) [28].

### Covariates

Covariates were measured using the most recent information available in CPRD GOLD prior to the date of osteoarthritis diagnosis, using a look-back period of up to 5 years. Demographics and clinical characteristics included age, sex, IMD quintile [29], BMI (< 18.5, 18.5–24.9, 25.0–29.9, 30.0–34.9, 35.0–39.9, and ≥ 40.0 kg/m^2^), smoking status (current, ex-, never) and alcoholic drinking status (current, ex-, never). Prior diagnoses and prescribed medications were also included, as listed in Additional File 2, as was prior referral to physiotherapy and Charlson comorbidity score (0, 1, 2 or ≥ 3) [30].

### Statistical analysis

**Fig. 1:**
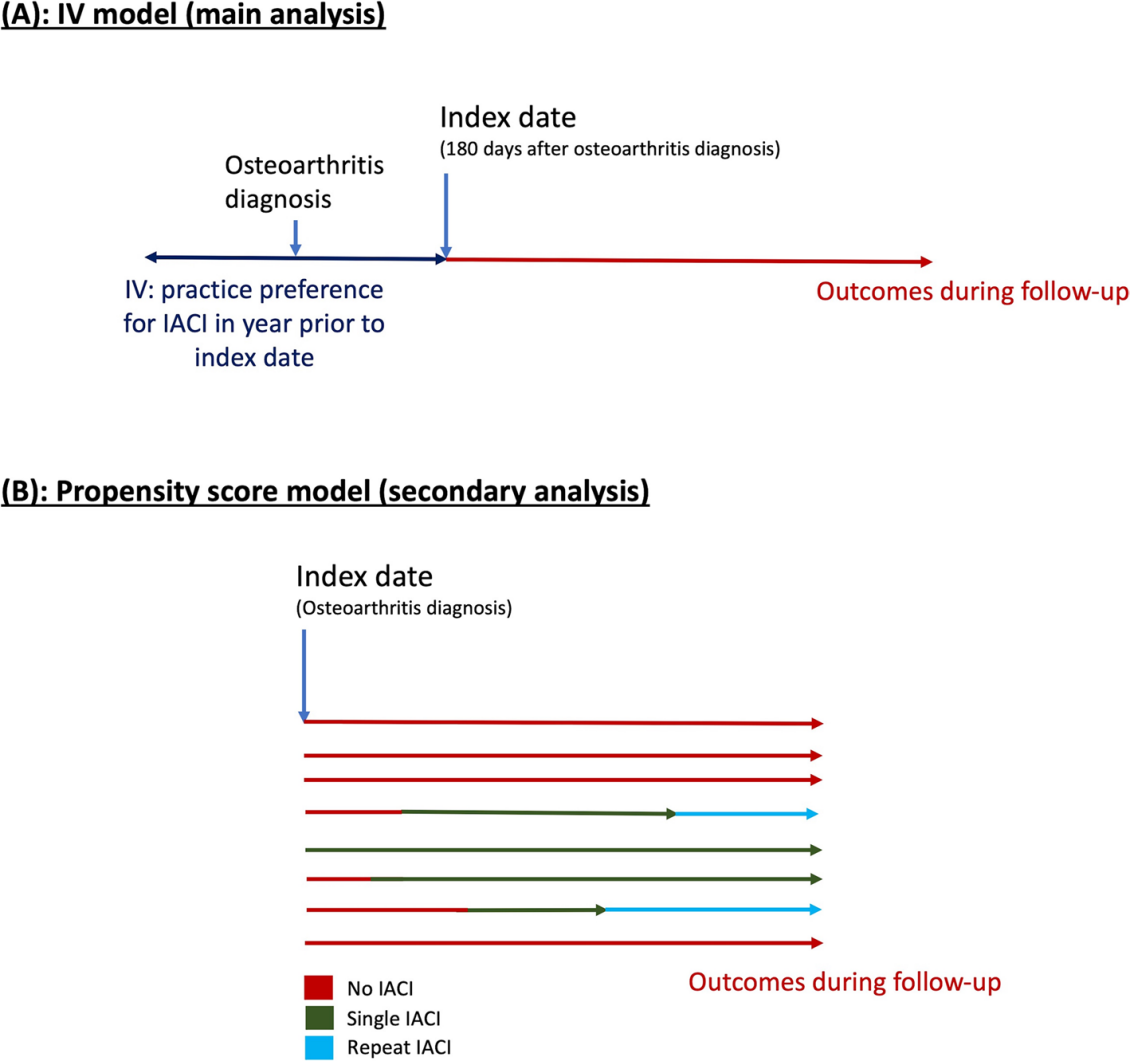
Diagram of model set-up for analyses estimating the effect of IACI on subsequent rates of joint surgery

Instrumental variable (IV) analysis was a priori chosen as the primary statistical approach. Reasons for this included the suspected strong confounding by indication in an investigation of the ‘real world’ effect of IACI on surgery. Important unmeasured confounders not available in the CPRD dataset would also otherwise be unaccounted for, such as pain and radiographic severity, rate of disease progression, physical function, and quality of life, amongst other factors. The IV approach can yield unbiased estimates of treatment effect in such situations, permitting key assumptions are met (the conceptual framework for the IV model is included in Additional File 1: Fig. S1) [31, 32]. The IV derived and used was a binary indicator of GP practice preference for IACI use during a 1-year exposure window around the date of osteoarthritis diagnosis (Fig. 1). Within this exposure window, GP practice preference was defined as IACI use over the previous 20 patients diagnosed with knee osteoarthritis at that practice being greater than the national median use in the sample [33]. Preference for single or repeated IACI use was considered separately, compared to non-use. Repeated use was defined as two or more injections. We did not analyse further granularity given the small number of patients receiving more than two injections (publication in-press). In line with assumptions of IV analysis, we a priori determined the method would only be used where the instrument was found to sufficiently predict treatment (odds ratio (OR) ≥ 2 and* F*-statistic > 10 [31, 33]) and where it was independent of measured potential confounders (standardized mean difference (SMD) ≤ 0.1 [34, 35]). We included a category for missingness in BMI and smoking/drinking status, and tested for balance in the IV across these missing categories. We only proceeded to use the method if we observed balance across missingness, as per the other potential confounders. Knee replacement was analysed at 1- and 5-year follow-up from model index date, defined as 6 months after osteoarthritis diagnosis (Fig. 1). Patients were followed up from index date until the earliest of the following: outcome date, death date, end of follow-up, or study end. Two-step Poisson regression models were used [33, 36], producing scaled incidence rate ratios (IRR) alongside 95% CI estimating the attributable effect of IACI use versus non-use.

In secondary analyses, the propensity score (i.e. individual conditional probability) of IACI use during follow-up was estimated for each patient using only the covariates available in the dataset. This propensity was used to match patients who received IACI to similar patients who did not receive IACI, using 1:2 greedy matching within a calliper width of 0.2 standard deviations (SD) [37]. Exact matching was implemented for geographic regions and calendar years to ensure variation associated with those factors was accounted for. All covariates listed above (described in Additional File 2, i.e. over 50 variables) were included in the logistic regression propensity score estimation. Multiple imputation using chained equations (MICE) was used to impute missing data on BMI, smoking, and drinking status—assuming this data was missing at random—as has been used previously [38]. A time-varying exposure approach was taken where follow-up from the osteoarthritis diagnosis date was split according to time-specific exposure status: no IACI, single IACI, or repeated IACI (Fig. 1). Incidence rates of outcomes with 95% CI were calculated within each exposure period. Association between IACI use and outcomes was estimated using Cox proportional hazards survival analysis using calendar time as the follow-up axis, producing hazard ratios (HRs). Results across imputed datasets were pooled using Rubin’s rules.

For analysis of post-operative outcomes following knee replacement, the IV approach was not used due to breach of the assumptions of the method—specifically several covariates were imbalanced across levels of the IV in this sub-group. Post-operative outcomes were therefore analysed using propensity score matching only. Complications were measured at 6 months and 1 year. Re-operations were measured at 1 and 5 years. Index date for these Cox models was the date of knee replacement, and the use of IACI was measured between the date of osteoarthritis diagnosis and any date prior to knee replacement surgery. Differences in 6-month post-operative PROMs were compared between IACI exposure groups using linear regression.

### Sensitivity analyses

Sensitivity IV analyses were run, firstly using a later index date of 1 year after the osteoarthritis diagnosis date (while still using a 1-year look-back exposure window) to explore treatment preference slightly later into the disease course. Secondly, adjusting for geographic region and calendar year of osteoarthritis diagnosis.

### Patient and public involvement

There has been patient and public involvement at all stages of this research. We discussed the study proposal and interim findings with members of the Patient Experience Partnership in Research group—an established forum within our research group consisting of eight service users with a range of musculoskeletal conditions, including osteoarthritis.

## Results

There were 33,357 patients retained in IV analyses estimating the effect of single IACI use on the rates of surgery at 1-year follow-up: 19,845 patients registered to a GP practice preferring non-use of IACI and 13,512 preferring single IACI around the time of patients’ diagnosis of knee osteoarthritis (Table 1 and Additional File: Fig. S2). Similar although slightly smaller sample-sized cohorts were derived for investigating repeat IACI use and on outcomes at 5-year follow-up. Selected characteristics are shown in Table 1.

**Table 1:**
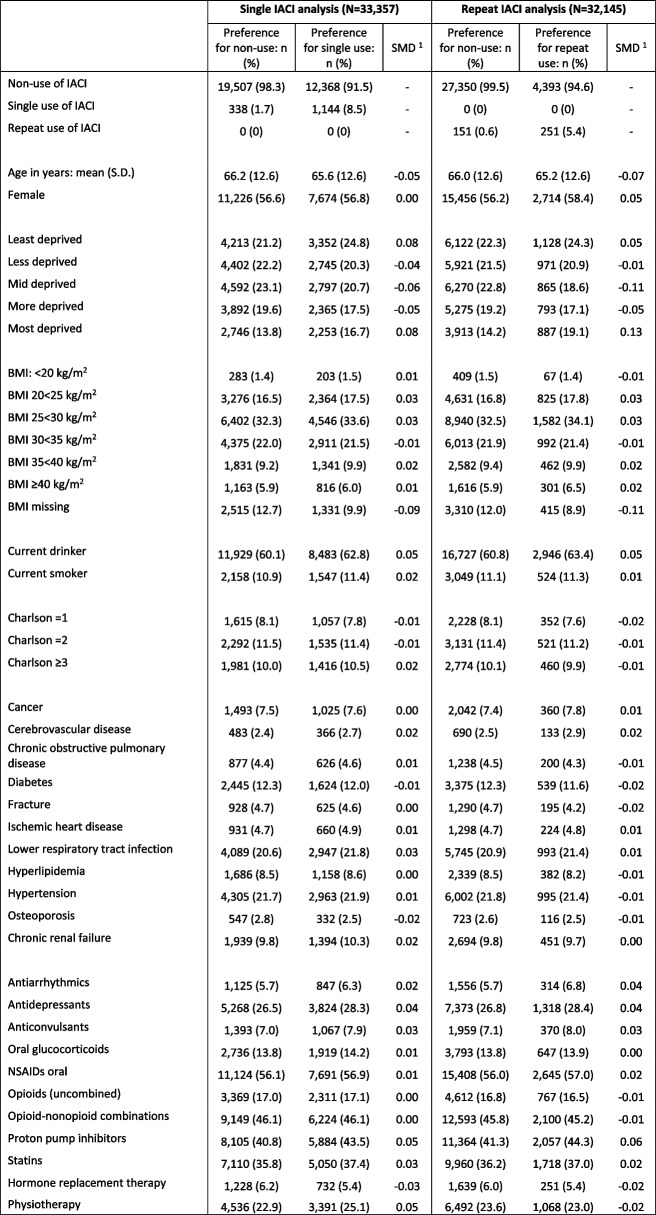
Selected characteristics of patients included in the main instrumental variable analysis estimating the effect of intra-articular corticosteroid injection (IACI) on subsequent rates of surgery

^1^Standardised Mean Difference (SMD) comparing baseline characteristics between IV groups (i.e. patients registered to a GP practice preferring IACI use compared to non-use around the time of patients’ index diagnosis). SMD values closer to zero indicate better balance and values greater than +/− 0.1 indicate non-balance

### IV diagnostics

The IV strongly predicted receipt of a single IACI treatment: OR = 5.34 (95% confidence interval (CI): 4.23, 6.73), *F* = 889.2 (*P* < 0.001). For illustration, 1144 (8.5%) received IACI amongst those whose GP practice preferred single IACI, whilst this number was just 338 (1.7%) for those whose GP practice preferred non-use of IACI (Table 1). For repeat IACI use, the corresponding OR was 10.35 (95% CI: 7.42, 14.42), *F* = 776.9 (P < 0.001), with the percentage of patients receiving repeat IACI being 5.4% vs. 0.6% within the two preference groups (Table 1).

Exploratory analyses indicated there were considerable differences in patient characteristics between those who received an IACI and those who did not, although these fully attenuated (including across data missingness categories) when compared across levels of GP practice preference for single IACI (Table 1). However, some significant differences in patient characteristics were observed between levels of the IV when analysing repeat IACI use (Table 1); therefore, subsequent IV analyses were only performed to investigate single IACI.

### IV analysis of surgery rates

At 1-year follow-up there were a total of 1628 knee replacements, increasing to 3730 at 5-year follow-up. GP practice preference for single IACI compared to not using IACI was associated with a slightly lower absolute risk of knee replacement at 5 years (Additional File: Table S3 and Fig. S3): 12.3% (27.4/1000 PYs) *vs.* 13.5% (30.3/1000 PYs). There was little difference in the incidence of arthroscopy or debridement between IV levels (Additional File: Table S3 and Fig. S3).

**Fig. 2:**
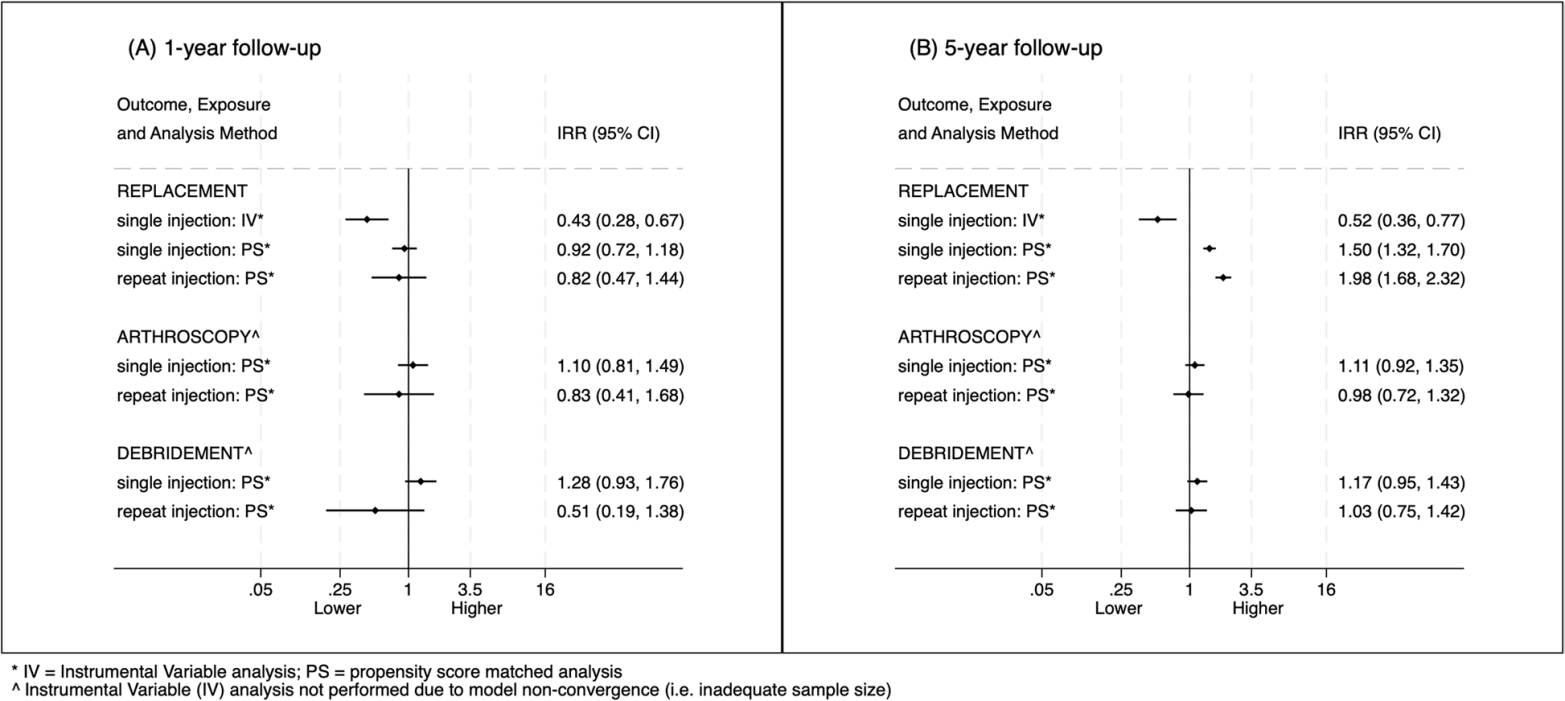
Estimated effect of IACI on subsequent occurrence of joint surgery analysed using instrumental variable and propensity score methods: **A** at 1-year follow-up and **B** 5-year follow-up

The primary two-step regression models would only converge for analyses of knee replacement, owing to too few outcome events in analyses of arthroscopy and debridement. Coefficients expressed as scaled IRRs suggested IACI to be attributable to an approximate halving in rates of knee replacement during either 1-year or 5-year follow-up (Fig. 2). To put such relative measures of association into context, were this a causal relationship then the number needed to treat (NNT) to avoid one occurrence of knee replacement at a 5-year follow-up would be 17 (95% CI: 12, 34) [39]. Findings were identical in sensitivity models adjusted for geographic region and calendar year (5-year IRR = 0.52, *p* < 0.001), and in those using a later index date (5-year IRR = 0.51, *p* = 0.03).

### Propensity score analysis of surgery rates

In secondary analyses accounting for measured covariates only, there were 6,425 knee osteoarthritis patients included, with good balance (SMD ≤ 0.1) demonstrated across all patient characteristics between IACI users and non-users (Additional File: Table S2). There were no significant differences in incidence rates of any surgical procedure within 1 year of IACI (Fig. 2A). Conversely, during 5-year follow-up rates of knee replacement were higher following a single (HR = 1.50 [95% CI: 1.32, 1.70]) or repeat (HR = 1.98 [95% CI: 1.68, 2.32]) IACI (Fig. 2B and Additional File: Table S4).

### Propensity score analysis of post-operative outcomes

Among 2828 included knee replacement patients, there were no significant differences in rates of post-operative complications or reoperations within any of the timeframes studied (Table 2 and Additional File: Fig. S4). Likewise, there were no clinically important differences in post-operative OKS [40], with mean values of 35.8 amongst non-injected, 36.0 for those who had received a single IACI (mean difference of 0.17 [95% CI: − 1.04, 1.38] for comparison to no IACI), and 36.8 for those who had repeated use of IACI (mean difference of 1.00 [95% CI: − 0.55, 2.54] for comparison to no IACI). Corresponding values for post-operative EQ-5-D were respectively 0.78, 0.77 (mean difference of − 0.01 [95% CI: − 0.03, 0.02] compared to no IACI) and 0.78 (mean difference of 0.00 [95% CI: − 0.04, 0.04] compared to no IACI).

**Table 2:**
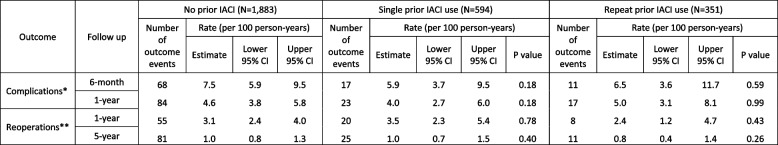
Estimated effect of intra-articular corticosteroid injection (IACI) on post-operative adverse events following knee replacement

*Complications: stroke, myocardial infarction, pulmonary embolism, deep vein thrombosis, and infection

**Reoperations: debridement, manipulation under anaesthetic, revision and other

## Discussion

### Main findings

In primary analysis of single IACI, we observed an associated reduction in subsequent occurrence of knee replacement aduring 5-year follow-up, although this was not replicated in secondary analyses in which conflicting results were observed. Reassuringly, IACI use among those eventually requiring knee replacement surgery did not adversely affect post-operative outcomes.

### Findings in context and possible mechanisms of effect

There is limited literature on the possible effect of IACI on the subsequent need for surgical intervention [16]. One previous study found that IACI use was associated with an approximate 6-month increase in the time from first clinic presentation to joint replacement surgery [41], and concluded there was little to be gained from using injections in osteoarthritis patients for whom joint replacement was already appropriately indicated, except to ‘bridge the gap’ by providing interim symptom relief. However, this prior study was unable to investigate the possible effect of IACI on the actual risk of joint replacement, given the lack of a control group.

Contributing to the IV-associated risk reduction in knee replacement at 1-year could be a short-term clinician/surgeon reticence to operate on those recently treated with IACI, given concerns around postoperative joint infection [18, 23], although this is unlikely to explain the reduction persisting up to 5 years. Joint replacement should be considered when joint symptoms are substantially impacting quality of life and where non-surgical management (including non-opioid analgesia) is ineffective or unsuitable [12]. It is therefore possible that IACI may have brought symptoms of pain and function back under control to an extent that non-surgical management was no longer deemed ineffective. Short-term pain relief could also provide a ‘window of opportunity’ in which to establish therapeutic exercise and dietary weight management/reduction, which are themselves effective core treatments for osteoarthritis [1, 42], and could potentially slow or arrest the progression of chronic pain leading to surgery.

However, our secondary analyses using propensity score matching did not replicate this protective effect. Likewise, a previous matched analysis found continuous use of IACI to be associated with an increased risk (HR > 3) of radiographic osteoarthritis progression [19], although Latourte et al. subsequently concluded from their cohort study that IACI conferred no significant risk increase of radiographic worsening or knee replacement, but that replication of findings was required in other cohorts [22]. A previous randomized controlled trial (RCT) suggested no effect of repeated IACI on pain reduction over a 2-year period, but one of greater cartilage loss (although between-group differences in cartilage loss were halved and no longer significant amongst the subset who completed the trial) [20].

In terms of post-operative outcomes, it was reassuring to find no association between IACI use prior to knee replacement surgery and rates of post-operative complications, re-operations, or PROMs. A recent meta-analysis on this topic reported a small and non-statistically significant increase in the risk of post-operative prosthetic joint infection following knee replacement associated with any IACI use prior to surgery, which became larger and statistically significant when the time interval between injection and surgery was less than 3 months [43]. Following exploratory analyses, we opted to use a composite outcome for complications owing to the small number of outcome events, however in a post-hoc analysis specifically of post-operative wound or prosthetic joint infections, there was no association between prior IACI use and incidence rates of infection (Additional File 1: Fig. S4). During 1-year follow-up, these rates (per 100 PYs) were: no IACI = 3.1 (95% CI: 2.4, 4.0), single IACI = 2.3 (95% CI: 1.3, 3.9), and repeat IACI = 3.9 (95% CI: 2.2, 6.6). We were unable to specifically investigate injections given less than 3 months before surgery owing to very few injections given within this timeframe, which reflects the existing caution in clinical practice given the potential risk of infection.

### Differences between primary and secondary analyses

The IV was a priori used as a primary analysis given it provides a stronger means for dealing with the inherent confounding by indication likely to be profoundly present in an investigation of the ‘real-world’ effectiveness of IACI. The method makes certain assumptions (Additional File: Fig. S1), but where fulfilled the method can enable the estimation of treatment effect in a manner unbiased by either strong, unmeasured or unknown confounding [31, 44]. Conversely, secondary propensity-score methods were not able to factor in differences in any covariates that were not available in the primary care database, and therefore likely to have been affected by residual confounding. This is in keeping with previous studies in other settings that have likewise reported protective treatment effects that only became apparent following the use of the IV approach to deal with unmeasured confounding [45].

There could have been some infringement on model assumptions such as the exclusion restriction (i.e. the instrument only being related to the outcome via the exposure, Additional File: Fig. S1), if clinician preference to recommend IACI were heavily influenced by local delays to surgery due to National Health Service (NHS) waiting lists. While this could potentially lead to over-estimation of the benefit of IACI, it’s worth noting no such delays were identified in qualitative interviews with GPs as a decision-making factor for administering IACI, [46] and would arguably not explain a reduction observed in knee replacement rates over the longer-term, i.e. up to 5 years. It is also very reassuring that we observed balance in all observed patient covariates, including deprivation levels, across IV groups for a single IACI, although we were nonetheless unable to confirm whether the instrument was truly exogenous as this is not empirically verifiable.

Also worth noting is that in main IV analyses, practice preference for IACI was estimated around the time of osteoarthritis diagnosis (where milder disease predicts stronger response to IACI [47]), whereas the propensity score analyses modelled exposure to IACI during 5 years follow-up. As such, the patient-level matching in these secondary models likely left them prone to time-varying confounding, given patients were matched only on baseline characteristics. This may explain why the secondary models only estimated an increase in knee replacement at 5-year rather than at 1-year follow-up (Fig. 2). In addition, the secondary analyses relied on a much smaller and less generalisable sample. Specifically, the estimates from the IV models have a similar interpretation to the ‘local average treatment effect’, which is the effect of IACI exposure among patients whose treatment status depended on the level of the instrument [32, 48], whereas the secondary analyses only estimated the average treatment effect in the treated population for whom there was propensity score overlap with those not receiving IACI. Further limitations to the propensity score matching method have previously been identified, including the lack of complete fully blocked randomization, reliance on assessing average levels of imbalance and concerns around model dependency possibly leading to bias [49].

### Strengths and limitations of the study

The CPRD GOLD dataset is a large and generalisable sample (on a national level), in terms of age, sex and ethnicity [25]. The longitudinal and linked nature of data meant longer-term outcomes could be reliably and efficiently ascertained. Whilst identification of osteoarthritis in the database has been validated, the timing of diagnosis in relation to disease onset may be less reliable [50]. We did not distinguish between primary and secondary osteoarthritis, nor were we able to perform external validation of code lists used to identify IACI. We also included IACI codes not specifying the joint site injected although we addressed this issue by excluding patients with osteoarthritis diagnosed at more than one anatomical joint site. Given the absence of laterality information in the database we were unable to confirm whether subsequent surgeries were at the same joint as that injected or the contralateral knee, which may have introduced some bias towards the null. We were also unable to distinguish IACI administered in secondary care which is more often performed using ultrasound or other imaging guidance.

### Clinical implications and future research

The findings from this work can be used to refine the use of IACI in the patient pathway and should facilitate the shared decision-making process between patients and clinicians when considering the use of IACIs for osteoarthritis. The reduction in rates of knee replacement following the use of IACI as observed in our IV analysis suggests a strategy of using IACI for knee osteoarthritis may be effective in helping manage uncontrolled pain, possibly conferring an appreciable delay to meeting the threshold for surgery. However, given our propensity score analyses did not replicate these findings but instead yielded conflicting results due to suspected residual confounding factors, future randomised studies are most likely needed to confirm and further elucidate the impact of single and repeat IACI use on the need for surgery. Unanswered questions include what the possible mechanisms of effect are, when in the osteoarthritis pathway patients should be offered IACI, what is the value for money, and what dose and type of IACI is most effective. The replication of the study in other healthcare settings elsewhere in the world is another subject for future research.

## Conclusions

Findings from our main IV analysis suggest that short-term pain reduction following IACI for knee osteoarthritis may translate to lower rates of knee replacement over 5 years follow-up, although contradictory associations were observed in secondary analyses which likely reflected residual confounding by indication. Reassuringly, IACI was not associated with post-operative adverse events.

## Disclaimer

This publication presents independent research commissioned by the National Institute for Health and Care Research (NIHR). The views and opinions expressed by authors in this publication are those of the authors and do not necessarily reflect those of the NHS, the NIHR, MRC, NIHR Coordinating Centre, the Health Technology Assessment programme or the Department of Health and Social Care.

## Supplementary Information

Additional file 1: Tables S1-S4 and Figures S1-S4. Table S1 – Read code terms used. Table S2 – Patient characteristics for propensity-score analyses. Table S3 – Description of outcome events in IV analysis. Table S4 – Description of outcome events in propensity score analysis. Figure S1 – Conceptual framework diagram for IV model. Figure S2 – Population flow diagram. Fig S3 – Cumulative incidence of outcomes in IV analysis. Fig S4 – Rates of individual post-operative complications and re-operations.

Additional file 2.

## Data Availability

Applications to access CPRD GOLD and linked data must be made directly to CPRD in accordance with CPRD’s Research Data Governance process.

## Abbreviations

APC: Admitted patient care
BMI: Body mass index
EQ-5-D: EuroQoL five dimensions
CI: Confidence interval
CPRD: Clinical Practice Research Datalink
GP: General practice
HES: Hospital Episode Statistics
HR: Hazard ratio
IACI: Intra-articular corticosteroid injection
IMD: Index of Multiple Deprivation
IRR: Incidence rate ratio
IV: Instrumental variable
LSOA: Lower-level Super Output Area
OHS: Oxford Hip Score
OKS: Oxford Knee Score
ONS: Office for National Statistics
OR: Odds ratio
NICE: National Institute for Health and Care Excellence
NNT: Number needed to treat
RCT: Randomised controlled trial
PROM: Patient-reported outcome measure
SMD: Standardised mean difference
UK: United Kingdom
