# Supplementary material for: Effect of intra-articular corticosteroid injections for knee osteoarthritis on the rates of subsequent knee replacement and post-operative outcomes: a national cohort study of England
Source: BMC Med. 2025 Apr 7;23:195. doi: 10.1186/s12916-025-04000-6 (PMC11974133; doi:10.1186/s12916-025-04000-6)
Supplement: Supplementary file 1 — Additional file 1: Tables S1-S4 and Figures S1-S4. Table S1 – Read code terms used. Table S2 – Patient characteristics for propensity-score analyses. Table S3 – Description of outcome events in IV analysis. Table S4 – Description of outcome events in propensity score analysis. Figure S1 – Conceptual framework diagram for IV model. Figure S2 – Population flow diagram. Fig S3 – Cumulative incidence of outcomes in IV analysis. Fig S4 – Rates of individual post-operative complications and re-operations. [file 12916_2025_4000_MOESM1_ESM.pdf]

Additional File 1: Supplementary Tables and Figures

Table S1: Read terms used to identify osteoarthritis cohorts and exposure to intra-articular corticosteroid injection	
**Readcode**	**Readterm**
**KNEE OSTEOARTHRITIS**	
N05z611	Knee osteoarthritis NOS
N053611	Patellofemoral osteoarthritis
N05zL00	Osteoarthritis NOS, of knee
N072100	Degenerative lesion of articular cartilage of knee
Nyu2511	[X] Unilateral primary gonarthrosis
N051B00	Primary gonarthrosis, bilateral
Nyu2500	[X]Other primary gonarthrosis
Nyu2811	[X] Unilateral secondary gonarthrosis
Nyu2800	[X]Other secondary gonarthrosis
Nyu2700	[X]Other secondary gonarthrosis, bilateral
N052A00	Post-traumatic gonarthrosis, bilateral
N052C00	Post-traumatic gonarthrosis, unilateral
N052600	Localised, secondary osteoarthritis of the lower leg
N05z600	Osteoarthritis NOS, of the lower leg
N051600	Localised, primary osteoarthritis of the lower leg
N053600	Localised osteoarthritis, unspecified, of the lower leg
N054600	Oligoarticular osteoarthritis, unspecified, of lower leg
N05zM00	Osteoarthritis NOS, of tibio-fibular joint
**INTRA-ARTICULAR CORTICOSTEROID INJECTION**	
7K6Z700	Injection of steroid into knee joint
7K6ZE00	Injection of Lederspan into knee joint
7K6ZB00	Injection of hydrocortisone acetate into knee joint
7K6ZJ00	Injection of steroid into hip joint
7K6Z200	Injection of therapeutic substance into joint
7K6Z300	Injection into joint NEC
7L19100	Injection of steroid for local action NEC
7L19000	Injection of triamcinolone for local action
7L19E00	Injection of triamcinolone
7K6ZK00	Intra-articular injection
7L11900	Ultrasound guided steroid injection

**Table S2: Selected characteristics of patients included in secondary propensity score analysis (N=6,425)**			
Variable	Non-IACI [1] use during follow-up (%)	Use of IACI [1] during follow-up (%)	SMD [2]
Age in years: mean (S.D.)	67.3 (12.2)	67.3 (12.3)	0.00
Female	59.7%	60.4%	0.00
Least deprived	24.0%	24.0%	0.00
Less deprived	21.6%	20.6%	0.00
Mid deprived	19.0%	20.8%	0.03
More deprived	17.2%	17.5%	0.00
Most deprived	18.1%	17.0%	-0.03
BMI: <20 kg/m^2	0.8%	0.7%	0.00
BMI 20<25 kg/m^2	18.7%	19.7%	0.01
BMI 25<30 kg/m^2	35.1%	35.5%	-0.01
BMI 30<35 kg/m^2	27.0%	26.3%	0.01
BMI 35<40 kg/m^2	11.8%	11.1%	-0.02
BMI ≥40 kg/m^2	6.5%	6.7%	0.00
Current drinker	78.0%	77.6%	0.00
Current smoker	11.1%	11.2%	-0.01
Charlson =1	7.9%	7.9%	0.00
Charlson =2	12.5%	12.4%	-0.02
Charlson ≥3	11.3%	12.5%	0.02
Cancer	8.5%	8.7%	0.01
Cerebrovascular disease	2.6%	3.2%	0.02
Chronic obstructive pulmonary disease	4.7%	5.1%	0.01
Diabetes	11.7%	12.1%	0.01
Fracture	5.5%	5.1%	-0.01
Ischemic heart disease	7.3%	6.4%	-0.01
Lower respiratory tract infection	25.0%	23.8%	-0.02
Hyperlipidemia	10.8%	10.0%	-0.01
Hypertension	22.4%	22.8%	0.00
Osteoporosis	2.8%	2.9%	0.00
Chronic renal failure	11.5%	12.4%	0.00
Antiarrhythmics	6.4%	6.5%	0.00
Antidepressants	33.3%	34.2%	0.00
Anticonvulsants	10.6%	11.5%	0.02
Oral glucocorticoids	16.8%	16.9%	0.00
NSAIDs oral	64.4%	64.4%	0.00
Opioids (uncombined)	24.3%	24.3%	0.00
Opioid-nonopioid combinations	57.0%	56.1%	-0.02
Proton pump inhibitors	50.3%	49.1%	-0.02
Statins	42.2%	40.8%	-0.02
Hormone replacement therapy	6.4%	7.2%	0.00
Physiotherapy	28.6%	29.0%	-0.02

[1] IACI: Intra-articular corticosteroid injection

[2] SMD: Standardised Mean Difference. SMD values closer to zero indicate better balance. Values greater than +/- 0.1 indicate non-balance.

Table S3: Description of outcome events in instrumental variable analysis

Procedure	Preference for non-use of IACI [1]				Preference for single IACI [1]			
	Number outcome events	Incidence rate (per 100 person years)			Number outcome events	Incidence rate (per 100 person years)		
		Estimate	lower 95% CI	upper 95% CI		Estimate	lower 95% CI	upper 95% CI
**1-year follow-up**								
Arthroscopy	475	2.4	2.2	2.6	369	2.7	2.4	3.0
Debridement	401	2.0	1.8	2.2	304	2.3	2.0	2.5
Replacement	1024	5.1	4.8	5.4	604	4.4	4.1	4.8
**5-year follow-up**								
Arthroscopy	952	1.2	1.1	1.3	607	1.2	1.1	1.3
Debridement	805	1.0	0.9	1.1	532	1.0	0.9	1.1
Replacement	2359	3.0	2.9	3.2	1371	2.7	2.6	2.9

[1] IACI: Intra-articular corticosteroid injection

Table S4: Description of outcome events in propensity-score matched analysis (n=6,425)

Procedure	Non-use of IACI [1]				Single use of IACI [1]					Repeat use of IACI [1]				
	Number outcome events	Incidence rate (per 100 person years)			Number outcome events	Incidence rate (per 100 person years)				Number outcome events	Incidence rate (per 100 person years)			
		Estimate	lower 95% CI	upper 95% CI		Estimate	lower 95% CI	upper 95% CI	P-value		Estimate	lower 95% CI	upper 95% CI	P-value
**1-year follow-up**														
Arthroscopy	139	4.7	3.9	5.5	62	5.1	3.8	6.3	0.56	8	3.9	1.2	6.6	0.60
Debridement	112	3.8	3.1	4.5	60	4.9	3.6	6.1	0.13	4	1.9	0.0	3.8	0.18
Replacement	217	7.4	6.4	8.4	88	7.1	5.7	8.6	0.52	13	6.3	2.9	9.7	0.50
**5-year follow-up**														
Arthroscopy	426	2.0	1.8	2.2	146	2.0	1.7	2.4	0.28	48	1.8	1.3	2.3	0.88
Debridement	366	1.7	1.5	1.9	134	1.8	1.5	2.2	0.14	45	1.6	1.1	2.1	0.83
Replacement	802	4.0	3.7	4.2	387	5.7	5.2	6.3	<0.001	192	7.6	6.5	8.7	<0.001

[1] IACI: Intra-articular corticosteroid injection

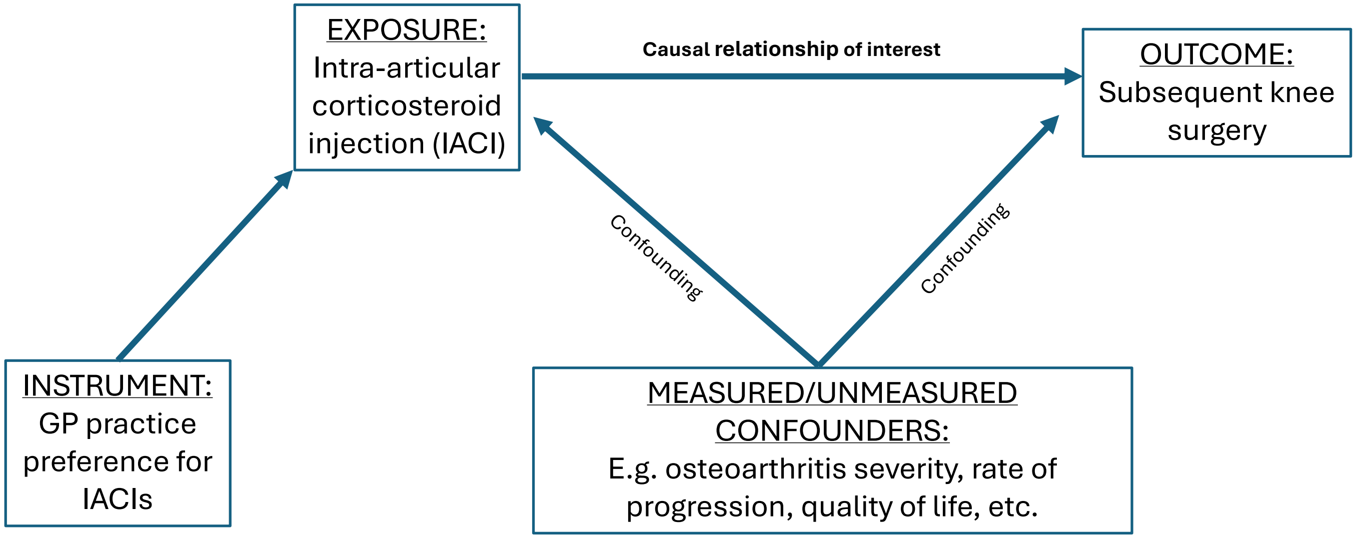

Figure S1: Conceptual framework illustrating main instrumental variable (IV) model. INSTRUMENT is used to isolate causal covariation in EXPOSURE -> OUTCOME, independent of measured/unmeasured confounded covariation. As such, the IV analysis estimates the causal effects of EXPOSURE on OUTCOME for people whose EXPOSURE is determined by the INSTRUMENT. Assumptions include that the INSTRUMENT predicts EXPOSURE, is not associated with CONFOUNDERS, and is not associated with OUTCOME except through the EXPOSURE.

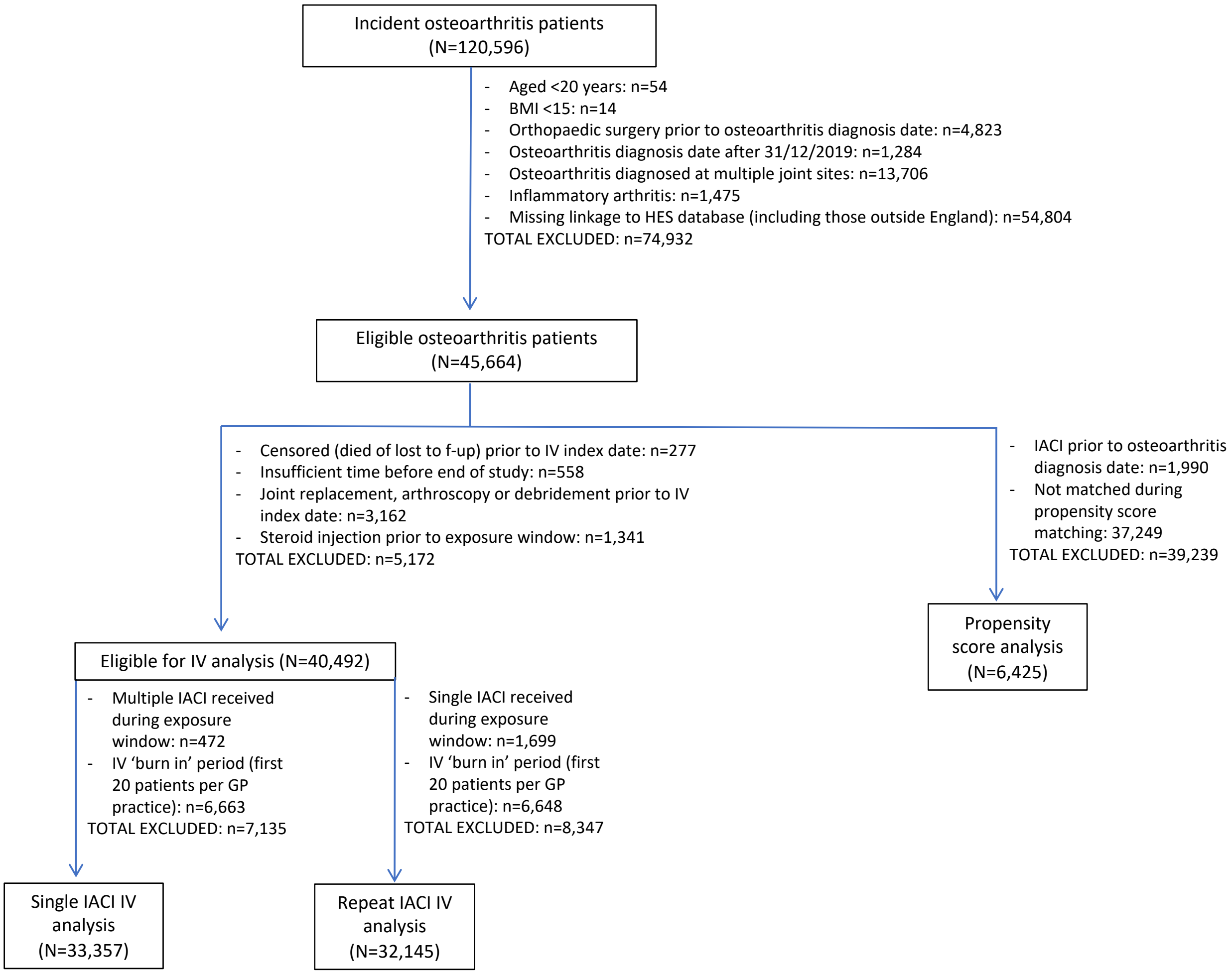

Figure S2: Population Flow Diagram

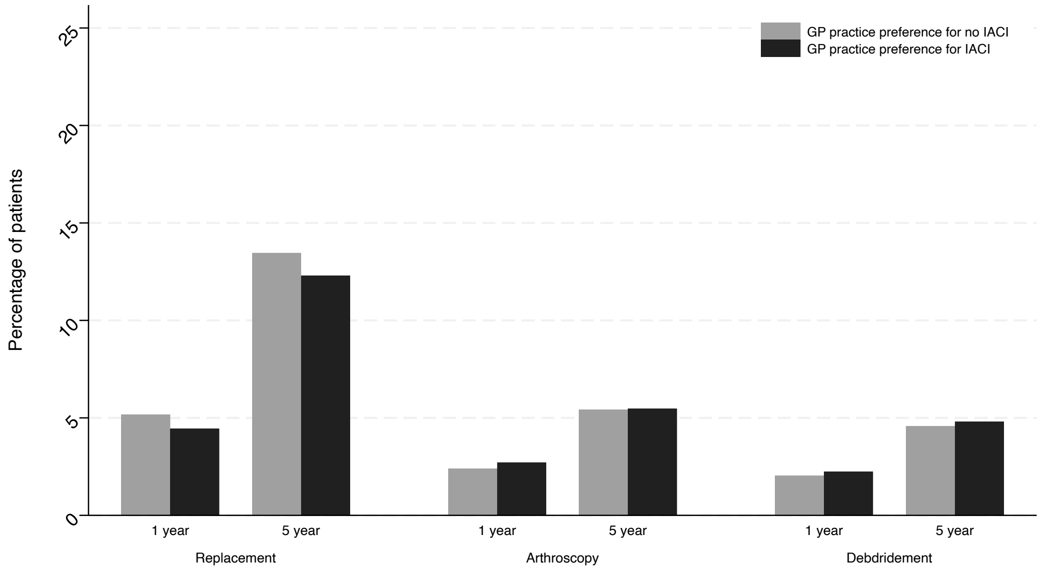

Figure S3: Crude cumulative incidence of outcome events during follow-up in Instrumental Variable (IV) analyses: stratified by preference for IACI

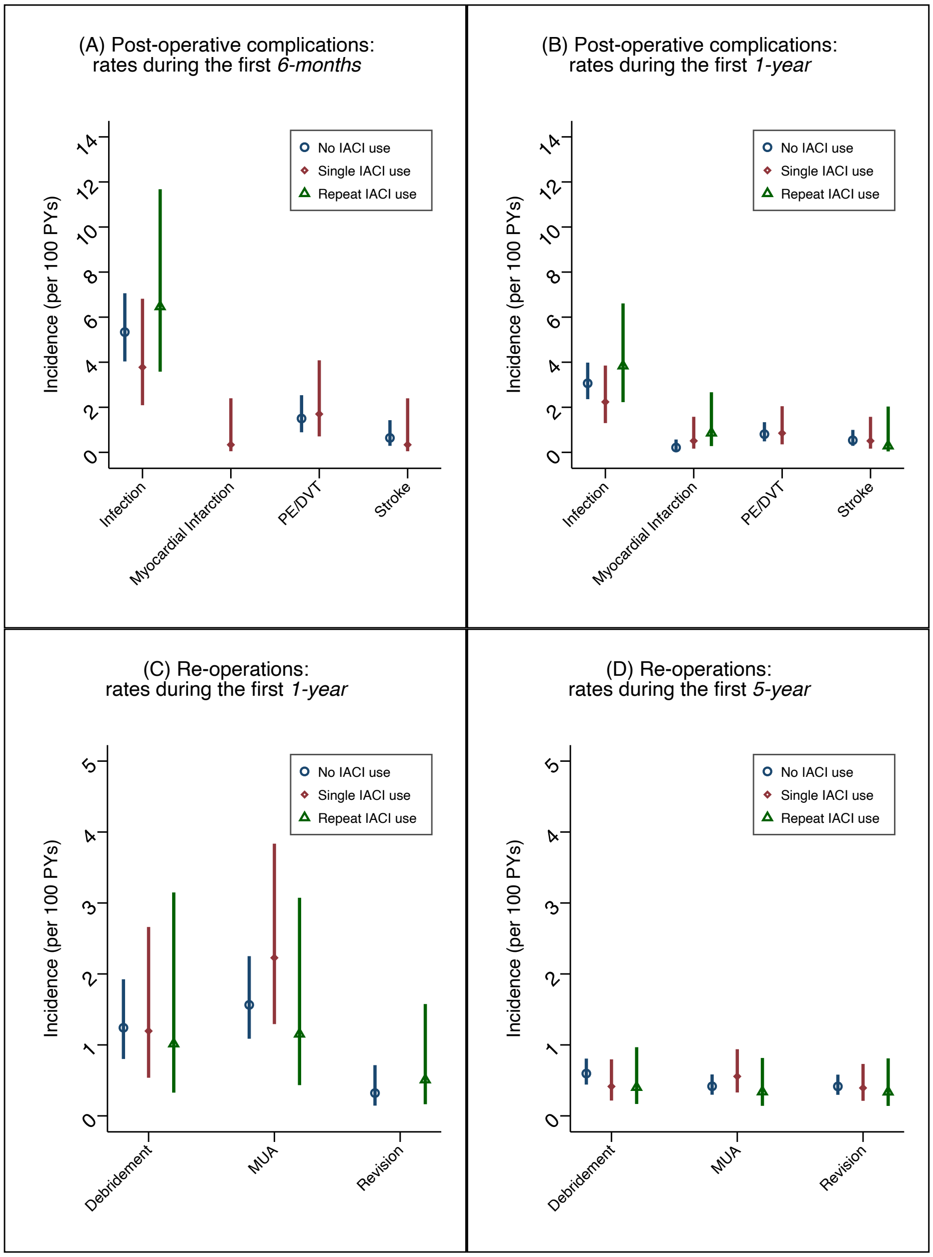

Figure S4: Incidence rates of individual post-operative complication and re-operation events during follow-up in propensity-score analyses
