# Supplementary material for: Effect of intra-articular corticosteroid injections for knee osteoarthritis on the rates of subsequent knee replacement and post-operative outcomes: a national cohort study of England
Source: BMC Med. 2025 Apr 7;23:195. doi: 10.1186/s12916-025-04000-6 (PMC11974133; doi:10.1186/s12916-025-04000-6)
Supplement: Supplementary file 2 — Additional file 2. [file 12916_2025_4000_MOESM2_ESM.pdf]

Additional File 2: List of previously diagnosed medical conditions and prescribed medications included in analyses

Prior diagnoses of the following medical conditions were also extracted from CPRD GOLD and included: asthma, cancer, chronic obstructive pulmonary disease (COPD), deep vein thrombosis, fracture, lower respiratory tract infection, myocardial infarction, pulmonary embolism, upper respiratory tract infection, urinary tract infection, anaemia, inflammatory arthritis, stroke, diabetes, epilepsy, ischemic heart disease, malabsorption, hyperlipidaemia, hypertension, osteoporosis, chronic renal failure and acute renal failure. Prior drug use was collected for the following medicines: antiparkinsonian medicines, antiarrhythmics, antibiotics, antidepressants, anticonvulsants, anxiolytics, bisphosphonates, calcium/vitamin D, oral corticosteroids, oral NSAIDs, topical NSAIDs, separate/loose opioids, opioid-nonopioid combinations, paracetamol, proton pump inhibitors, prednisolone, statins, hormone replacement therapy, or diuretics.
